# Intra-articular administration of IκBα kinase inhibitor suppresses mouse knee osteoarthritis via downregulation of the NF-κB/HIF-2α axis

**DOI:** 10.1038/s41598-018-34830-9

**Published:** 2018-11-07

**Authors:** Yasutaka Murahashi, Fumiko Yano, Hiroshi Kobayashi, Yuma Makii, Kousuke Iba, Toshihiko Yamashita, Sakae Tanaka, Taku Saito

**Affiliations:** 1 Sensory & Motor System Medicine, Graduate School of Medicine, The University of Tokyo, 7-3-1 Hongo, Bunkyo-ku, Tokyo, 113-8655 Japan; 2 Bone and Cartilage Regenerative Medicine, Graduate School of Medicine, The University of Tokyo, 7-3-1 Hongo, Bunkyo-ku, Tokyo, 113-8655 Japan; 3 Department of Orthopaedic Surgery, Sapporo Medical Univ. School of Medicine, S-1, W-16, Chuo-ku, Sapporo, 060-8543 Hokkaido, Japan

## Abstract

Activation of NF-κB signaling promotes osteoarthritis (OA) through the transcriptional induction of Hif-2α and catabolic enzymes. This study sought to examine whether inhibiting IκBα kinase (IKK) could suppress the development of surgically-induced OA of the knee in a mouse model. We employed BMS-345541 (4(2′-aminoethyl) amino-1, 8-dimethylimidazo (1,2-a) quinoxaline) as a selective inhibitor of the subunits of IKK. OA was created by resecting the medial collateral ligament and the medial meniscus in the knees of mice. The mice were then treated with an intra-articular injection of BMS-345541 (50 nM to 500 µM) or vehicle three times a week for 8 weeks. We found that the intra-articular administration of 500 nM and 5 µM BMS-345541 significantly suppressed OA development. In the BMS-345541-treated cartilage, there was a decrease in the phosphorylation of IκBα and the expression of Hif-2α, Mmp13, and Adamts5. In human articular chondrocytes, the IL-1β-enhanced expression of Hif-2α and catabolic factors were decreased by BMS-345541 treatment in dose-dependent manner. We conclude that the intra-articular administration of BMS-345541 at some concentrations may suppress the development of OA by downregulating signaling through the NF-κB–Hif-2α axis.

## Introduction

Osteoarthritis (OA) is the most common joint disorder in older people. Although joint pain can be partially controlled, the development of OA cannot be prevented, and degenerated articular cartilage cannot be regenerated by current therapeutics.

Since the development of surgically-induced mouse models of OA, various molecules and signaling pathways have been shown to be involved in its pathophysiology^1,2^. Among them, hypoxia-inducible factor-2a (Hif-2α) plays essential roles in OA development through the transcriptional induction of matrix metallopeptidase 13 (Mmp13), a representative cartilage-degrading enzyme^3,4^. Recently, we revealed that the activity of the nuclear factor kappa-B (NF-κB) signaling pathway is also tightly associated with the development of OA, along with other factors upstream of Hif-2α^5^.

The NF-κB family includes v-rel reticuloendotheliosis viral oncogene homolog A (Rela, also known as p65), Relb, Rel, p105/p50 and p100/p52. Inhibitors of NF-κB (IκB) proteins bind to some NF-κB family members in the cytoplasm^6^. Following the activation of IκB kinases (IKKs) in response to several signals, IκB proteins are phosphorylated and degraded. This frees NF-κB complexes and permits their translocation from the cytoplasm into the nucleus, leading to target gene transactivation^7,8^. In articular cartilage, IκBα is phosphorylated and Rela is translocated into the nucleus during OA development^5^. Intriguingly, studies have shown that homozygous knockout of Rela in adult chondrocytes accelerates OA due to enhanced chondrocyte apoptosis, whereas heterozygous knockout suppresses OA development through a downregulation of Hif-2α and Mmp13^5^. Studies in mouse primary chondrocytes have confirmed that Rela is indispensable for chondrocyte survival via the transcriptional induction of anti-apoptotic genes, and that activation of NF-κB signaling leads to induction of Hif-2α and catabolic factors^5^. Thus, NF-κB signaling must be tightly regulated for articular cartilage homeostasis and the prevention of OA.

Here, we employed BMS-345541 (4(2′-aminoethyl) amino-1, 8-dimethylimidazo (1,2-a) quinoxaline) as a selective inhibitor of the subunits of IKK^9^, and examined whether inhibiting IKK could suppress OA development *in vivo*. We performed intra-articular injections of BMS-345541 for 8 weeks into the knee joints of mice surgically treated to induce OA, and then histologically analysed the development of OA and changes in the expression of OA-related proteins. We further examined the suppressive effects of the NF-κB–Hif-2α axis by BMS-345541 in human primary articular chondrocytes.

## Results

### Suppression of OA development by intra-articular injection of BMS-345541

**Figure 1:**
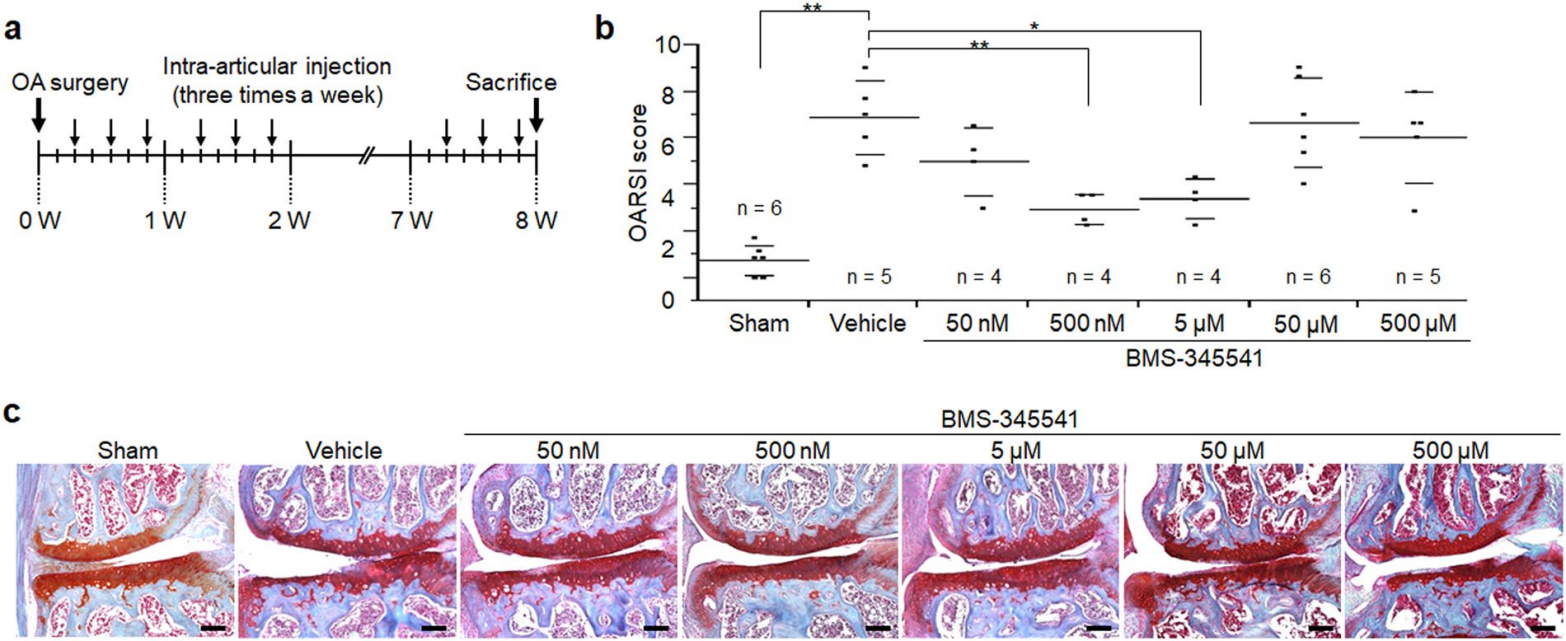
Effects of intra-articular administration of BMS-345541 in surgically-induced mouse knee osteoarthritis (OA). (**a**) Schematic representation of the surgical procedure for OA induction, intra-articular injections, and time of sacrifice. Injections were performed three times a week after surgery for 8 weeks. (**b**) Quantification of OA development using the Osteoarthritis Research Society International (OARSI) score for the first experiment. Symbols represent individual mice; long and short bars show the mean and SD, respectively. *P < 0.05, **P < 0.005 vs vehicle by ANOVA followed by Dunnett’s *post hoc* test. (**c**) Representative safranin O staining from each experimental group. Scale bar, 100 µm.

**Figure 2:**
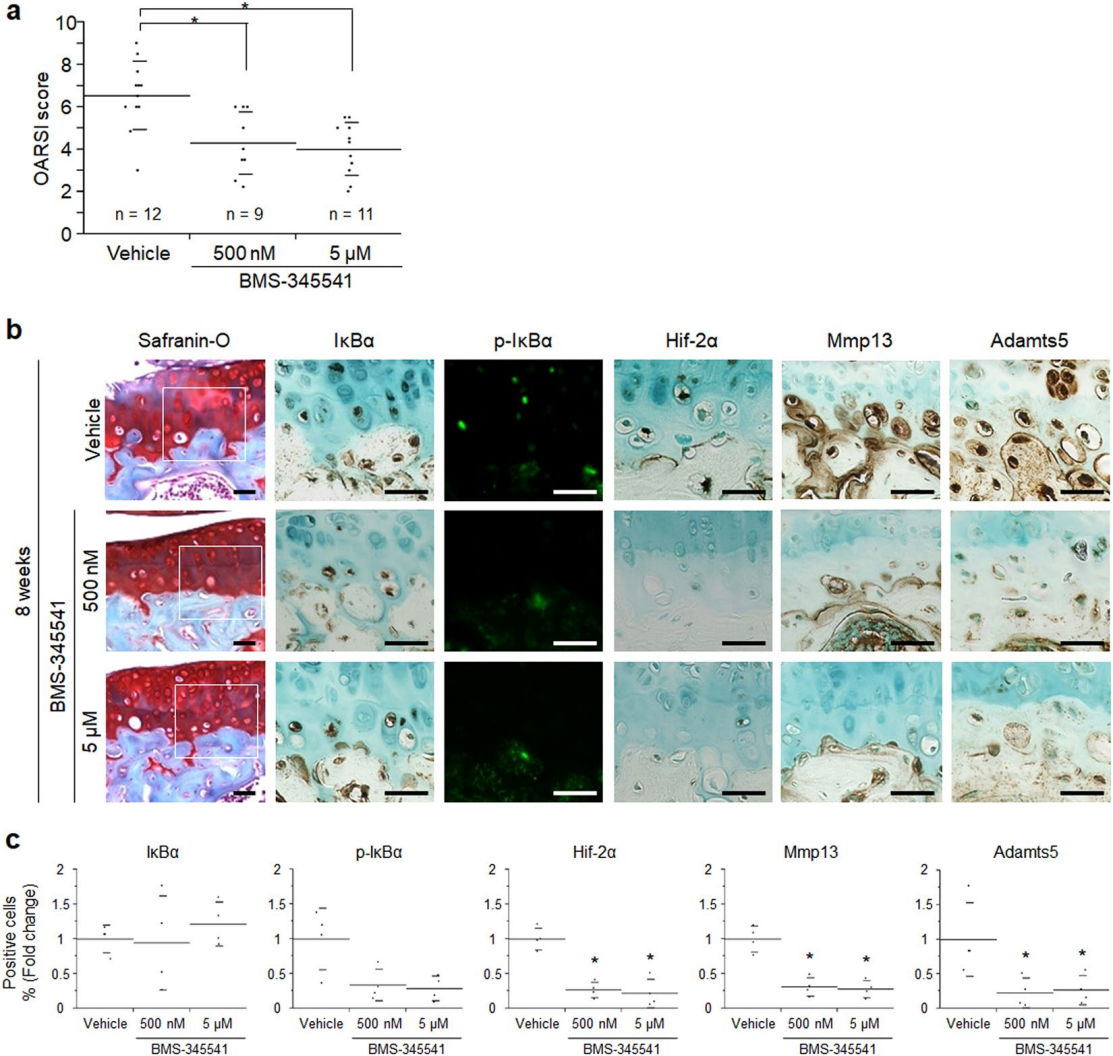
Suppression of the NF-κB–Hif-2α pathway by BMS-345541 treatment. (**a**) Quantification of OA development using the Osteoarthritis Research Society International (OARSI) score from the follow-up experiment as a confirmatory phase. Symbols represent individual mice; long and short bars show the mean and SD, respectively. *P < 0.005 vs vehicle by ANOVA followed by Dunnett’s *post hoc* test. (**b**) Safranin O staining and immunohistochemistry of NF-κB-related proteins and catabolic enzymes in the BMS-345541-treated cartilage at 8 weeks after surgical induction of OA. Inset boxes in safranin O staining indicate enlarged images. Scale bar, 50 µm. (**c**) The percentage of positive cells according to immunohistochemistry. *P < 0.05 vs vehicle by the Kruskal–Wallis test and Dunn’s *post hoc* test for unequal variance.

We first examined the effects of BMS-345541 in a surgically-induced mouse OA model. Periodic injections of BMS-345541 or HBC (vehicle control) were administered into the knee joints of mice three times a week for 8 weeks after surgery (Fig. 1a). The OARSI scores of the 500 nM and 5 µM BMS-345541 groups were lower in the first examination (Fig. 1b,c), so we additionally replicated treatment with intra-articular injections of vehicle, 500 nM and 5 µM BMS-345541 (Fig. 2a). Histological analyses and OARSI grading at 8 weeks after surgery revealed that the joints treated with 500 nM and 5 µM of BMS-345541 showed significantly less OA as compared with the vehicle control group (Fig. 2a), and the responsiveness was excellent (effect size, 1.509, 1.841; 95% confidence interval, 0.876–2.143 and 1.278–2.403 for 500 nM and 5 µM BMS-345541, respectively). The OARSI scores decreased in the animals in a dose-dependent manner up to 5 µM; however, its protective effect was not observed over 50 µM (Fig. 1b,c). Over the 8 weeks, we found no obvious alteration in body weight among the six groups (Supplementary Table S1).

### Suppression of the NF-κB–Hif-2α pathway by BMS-345541 treatment

**Figure 3:**
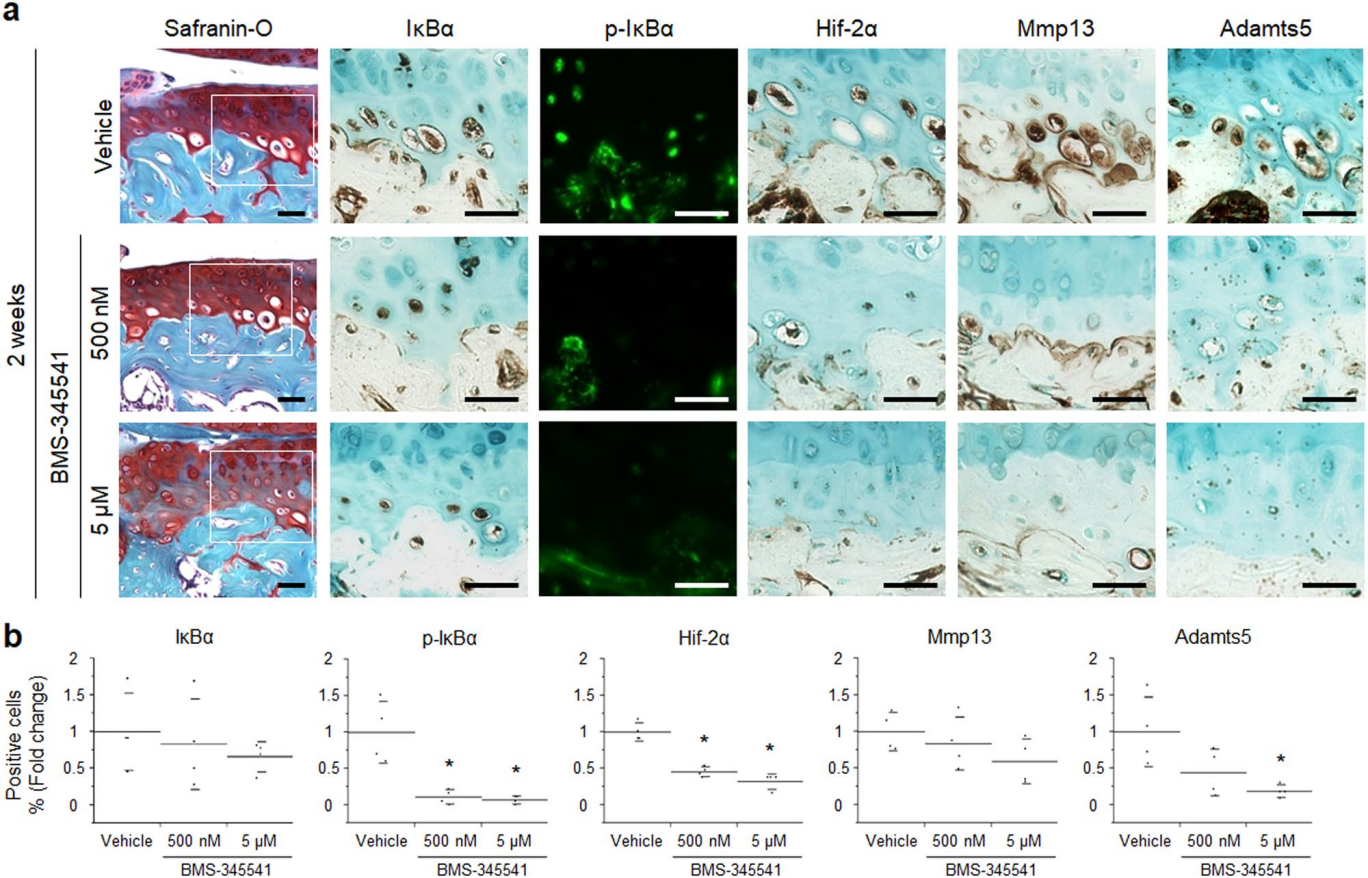
Effects of intra-articular administration of BMS-345541 in early phase of OA. (**a**) Safranin O staining and immunohistochemistry of NF-κB-related proteins and catabolic enzymes in the BMS-345541-treated cartilage at 2 weeks after surgical induction of OA. Inset boxes in safranin O staining indicate enlarged images. Scale bar, 50 µm. (**b**) The percentage of positive cells in the immunohistochemistry. *P < 0.05 vs vehicle by the Kruskal-Wallis test & Dunn’s post hoc test for unequal variance, n = 4 mice per group.

We next analysed the expression of NF-κB-related proteins and catabolic enzymes by immunohistochemistry. At 8 weeks, in addition to Hif-2α, the expression of Mmp13 and Adamts5 was decreased significantly in BMS-345541-treated cartilage (Fig. 2b,c). To examine the activity of NF-κB at an early stage of OA, we additionally prepared 4 mice per group, and performed histological analyses after 2 –weeks of BMS-345541 treatment. Although OA development was mildly observed at 2 weeks after surgery, there was no significant difference between any of the groups (Supplementary Fig. S1). Phosphorylation of IκBα was markedly suppressed in the BMS-345541-treated cartilage, as was the expression of Hif-2α at 2 weeks (Fig. 3a,b).

### Effects of BMS-345541 in human primary articular chondrocytes

**Figure 4:**
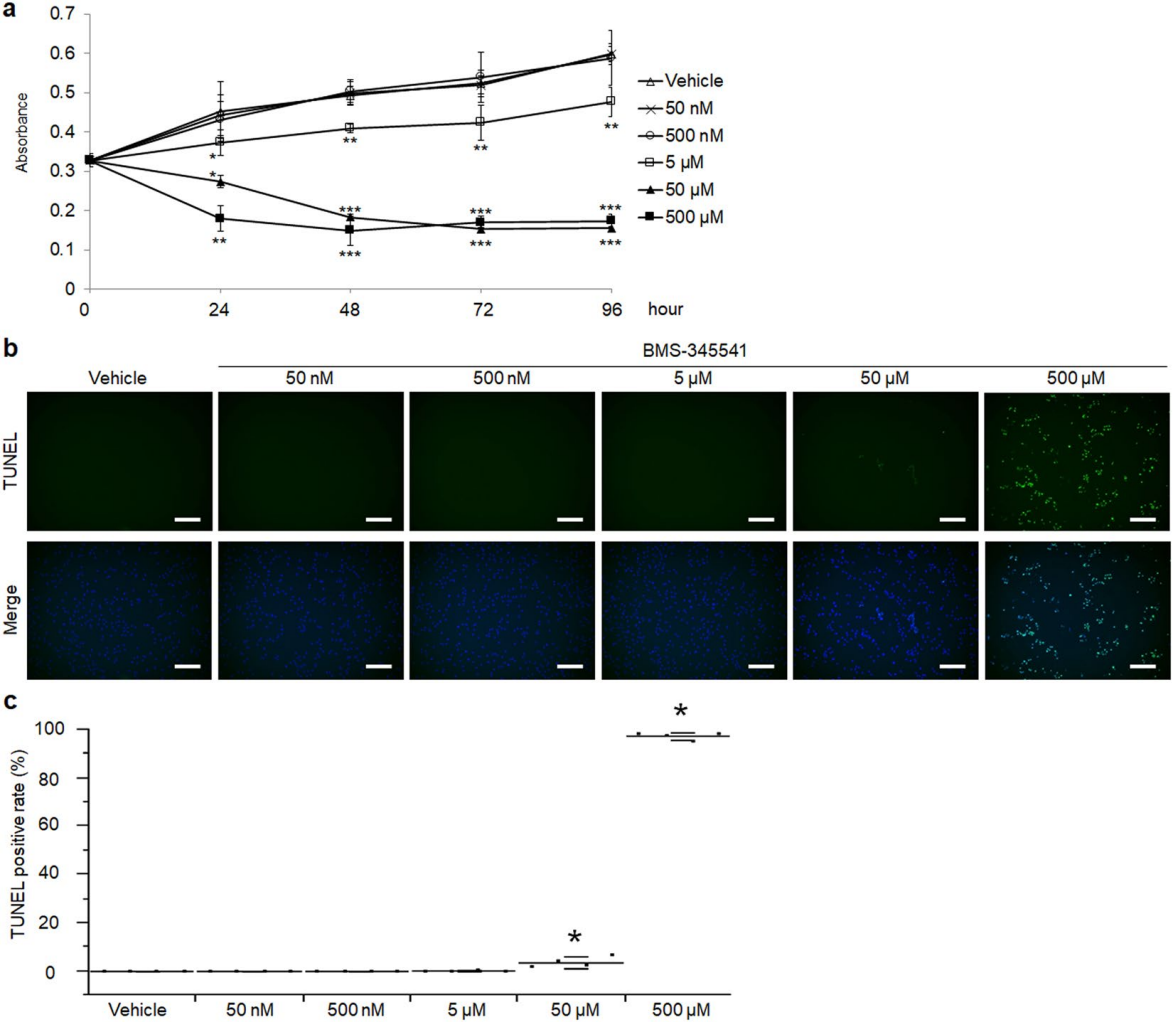
Proliferation and apoptosis of human articular chondrocytes treated with BMS-345541. (**a**) CCK-8 assay of human primary articular chondrocytes treated with different concentrations of BMS-345541 with or without 10 ng/ml IL-1β. Data are expressed as the mean of three wells per group. Error bars indicate 95% confidence interval. *P < 0.01, **P < 0.001, ***P < 0.0001 vs vehicle at each time point by the Kruskal–Wallis test and Dunn’s *post hoc* test for unequal variance. TUNEL stainings (**b**) and the rates of TUNEL positive cells (**c**) of human articular chondrocytes treated with vehicle or 50 nM, 500 nM, 5 µM, 50 µM, 500 µM BMS-345541 for 12 hours. Scale bars, 200 µm. Symbols represent individual points; long and short bars show the mean and SD of four slides per group, respectively. *P < 0.05 vs vehicle by Kruskal-Wallis test & Dunn’s post hoc test for unequal variance.

To examine the molecular mechanisms underlying the cartilage protective effect of BMS-345541, we performed *in vitro* experiments using human articular chondrocytes derived from surgical specimens. We first examined the effects of BMS-345541 on cell proliferation and apoptosis. Cell proliferation was not affected by BMS-345541 concentrations of 500 nM or lower, but was moderately decreased by 5 µM BMS-345541, and decreased by concentrations over 50 µM (Fig. 4a). Most chondrocytes died upon treatment with 50 µM or 500 µM BMS-345541 until 48 h (Supplementary Fig. S2), and TUNEL staining indicated significantly enhanced apoptosis upon treatment with 50 µM or 500 µM BMS-345541 (Fig. 4b,c).

**Figure 5:**
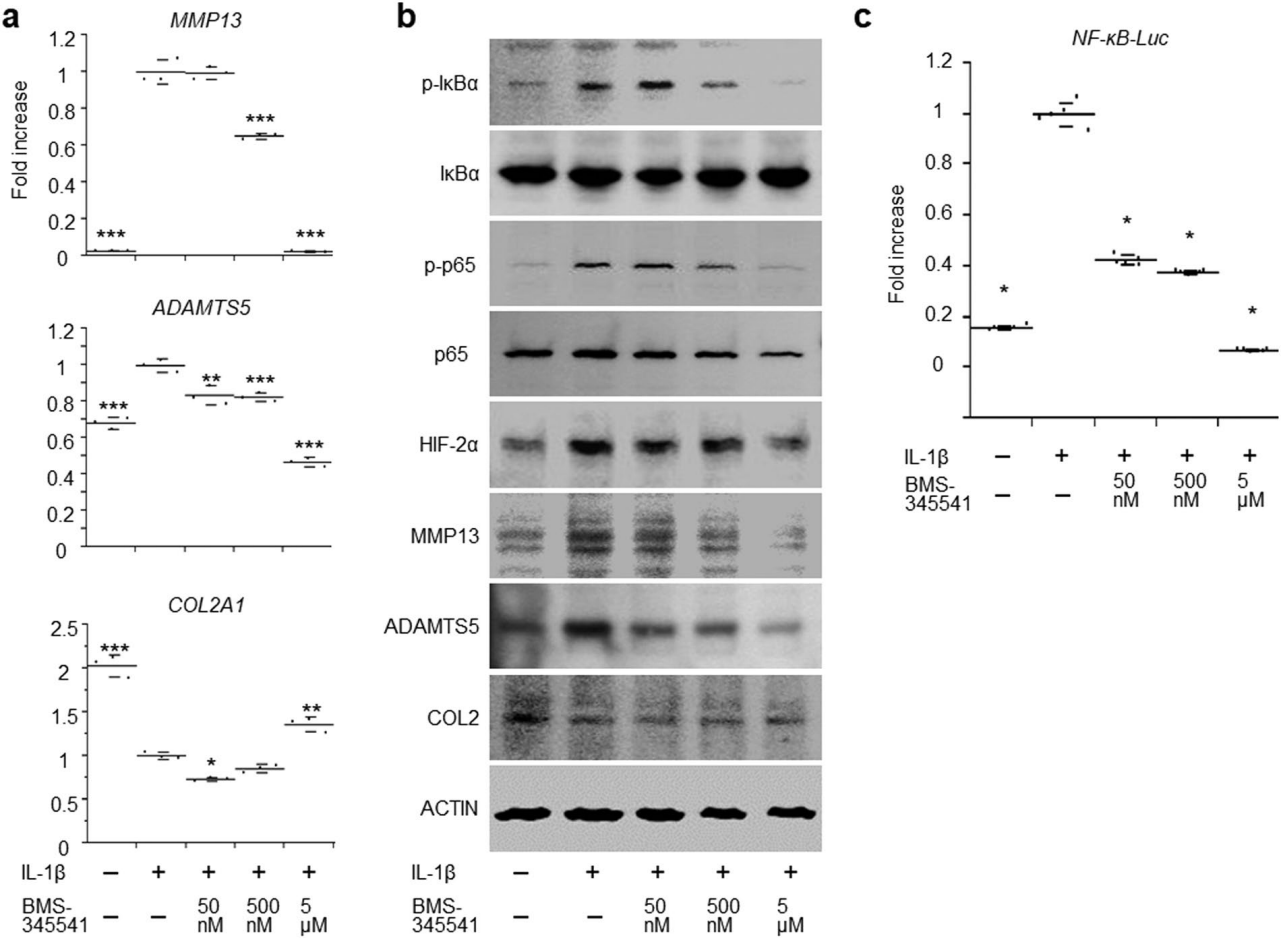
Effects of BMS-345541 in cultured human articular chondrocytes. (**a**) mRNA levels of *MMP13*, *ADAMTS5*, and *COL2A1* in human primary articular chondrocytes treated with different concentrations of BMS-345541 with or without 10 ng/ml IL-1β. BMS-345541 and IL-1β were added simultaneously, and the cells were cultured for an additional 24 h. The cells obtained from the three individuals showed similar responses, and a representative result is shown. Symbols represent individual points; long and short bars show the mean and SD of three wells per group, respectively. *P < 0.01, **P < 0.001, ***P < 0.0001 vs IL-1β+/BMS-345541− by the Kruskal–Wallis test and Dunn’s *post hoc* test for unequal variance. (**b**) Immunoblot analyses of human primary articular chondrocytes treated simultaneously with different concentrations of BMS-345541 with or without 10 ng/ml IL-1β. (**c**) Luciferase activities in human articular chondrocytes transfected with the NF-κB reporter vector. BMS-345541 was added 24 h after transfection, and the cells were cultured for an additional 24 h. Symbols represent individual points; long and short bars show the mean and SD of five wells per group, respectively. *P < 0.0001 vs IL-1β+/BMS-345541− by the Kruskal–Wallis test and Dunn’s post hoc test for unequal variance.

To examine whether BMS-345541 exerts a suppressive effect against the NF-κB–Hif-2α pathway in humans as well as in mice, the cells were treated with IL-1β with or without BMS-345541. We found that the induction of catabolic enzymes including *MMP13* and *ADAMTS5* by IL-1β was could be suppressed by BMS-345541 treatment in a dose-dependent manner, when administered simultaneously or at 3 hours after IL-1β stimulation (Fig. 5a,b, Supplementary Fig. S3). Alterations to the expression of COL2A1, a representative cartilage matrix protein, were not consistent (Fig. 5a,b, Supplementary Fig. S3). The phosphorylation of IκBα and Rela, and the increase in HIF-2α expression in IL-1β-treated cells could be inhibited by 5 µM BMS-345541 (Fig. 5b). Finally, using the NF-κB reporter luciferase construct, we confirmed that BMS-345541 suppressed the activation of NF-κB by IL-1β in a dose-dependent manner (Fig. 5c).

## Discussion

Here, we showed that the intra-articular administration of an IKK inhibitor could regulate the development of surgically-induced OA of the knee in mice. Histological analyses indicated that the activity of NF-κB was decreased in articular cartilage treated with the IKK inhibitor before OA development, and that the subsequent induction of catabolic factors was also decreased. In addition, we showed that similar chondro-protective effects were observed in human primary articular chondrocytes treated with the IKK inhibitor. OA was most effectively suppressed by 500 nM and 5 µM BMS-345541, meanwhile higher doses of BMS-345541 showed cytotoxity determined by the CCK-8 and apoptosis assays.

BMS-345541 was developed and first reported by Bristol-Myers Squibb Pharmaceutical Research Institute in 2003^9^. It has been widely used as a highly selective inhibitor of IKK in many experimental studies. McIntyre and colleagues previously reported that the oral administration of BMS-345541 is efficacious against collagen-induced arthritis (CIA) in mice^10^. Meanwhile, 15 years has passed since the first publication with this compound, and there is almost no possibility for its clinical applicability. In the present study, we selected the compound among several IKK inhibitors to examine whether OA could be suppressed by IKK inhibition, because the selectivity and efficacy of the compound have been confirmed by many previous studies^9–16^. Although the compound will not be used clinically, the present data indicates that IKK may be a potent therapeutic target to suppress OA via inhibition of the IKK-NF-κB-HIF-2α axis.

Although IKK inhibition may be useful for the prevention or treatment of various diseases, including OA, the side effects of such inhibition should be carefully examined for its clinical utility, because the IKK-NF-κB pathway is an important pathway in numerous cellular processes, such as cell adhesion, differentiation, proliferation, autophagy, senescence, and apoptosis^17^. According to a previous study^10^, mice treated with BMS-345541 perorally daily for 6 weeks showed no toxicological changes in their vital organs on gross or histological observation, even at higher doses of 100 mg/kg/day, which is about 10^6^ times the amount of BMS-345541 used in the present study. However, Yang *et al*. showed a 14% loss of body weight in melanoma-inoculated nude mice receiving high doses of oral BMS-345541 (75 mg/kg/day)^13^. In the present study, we could suppress OA development using a localized delivery of a much lower amount of BMS-345541 without causing body weight changes or obvious health impairment. The IKK inhibitor may be better suited to treating OA rather than systemic inflammatory joint diseases, because OA usually occurs in selective joints that can be independently targeted. However, in addition to efficacy, safety should be carefully examined when we target the IKK-NF-κB pathway through a novel approach.

In conclusion, the intra-articular administration of BMS-345541 at some concentrations may suppress the development of surgically-induced mouse knee OA through downregulation of the NF-κB–HIF-2α signaling axis. This inhibitory effect is also replicated in human cells *in vitro*. These results indicate the potential beneficial effects of IKK inhibitors for the prevention of OA development, assuming that treatment regimens adhere to suitable lower doses and localized modes of delivery.

## Methods

### Ethics statement

We performed all animal experiments according to a protocol approved by the animal care and use committee of the University of Tokyo. We obtained samples of human articular cartilage from three individuals undergoing total knee arthroplasty with written informed consent, as approved by the Ethics Committee of the University of Tokyo. All methods were performed in accordance with the relevant guidelines and regulations.

### Mice

Male C57BL/6J mice were housed in plastic cages with sawdust bedding in a specific pathogen-free facility, with 4 to 5 animals per cage. The room was maintained under a 12-hour light/dark cycle and at a constant temperature (18 °C to 23 °C). Mice could move freely in the cages and had free access to food and water.

### Osteoarthritis experiments *in vivo* and intra-articular administration

Experimental OA was induced in 8-week-old male mice, as described previously^2^. Briefly, under general anaesthesia, the medial collateral ligament and the medial meniscus were resected under a surgical microscope. For intra-articular injections, various concentrations (50 nM, 500 nM, 5 µM, 50 µM, 500 µM) of BMS-345541 (Sigma-Aldrich; St. Louis, MO, USA) were dissolved in saline (sterile 0.9% NaCl; Otsuka Pharmaceutical Factory, Naruto, Japan) containing 45% 2-hydropropyl-b-cyclodextrin (HBC; Sigma-Aldrich). The molecular weight of BMS-345541 is 291.78, and the actual weights of the administered drugs were 146 pg, 1.46 ng, 14.6 ng, 146 ng and 1.46 µg per 10 µL for 50 nM, 500 nM, 5 µM, 50 µM, and 500 µM, respectively. Control mice received HBC in saline. To examine the effective doses of BMS-345541, we first performed OA surgery for 6 mice per group, and treated them with intra-articular injections (10 µL) of HBC in saline (vehicle) or 50 nM, 500 nM, 5 µM, 50 µM, 500 µM BMS-345541 three times a week for 8 weeks after surgery (Fig. 1a). OA severity was assessed at 8 weeks after surgery. The Osteoarthritis Research Society International (OARSI) scores of the 500 nM and 5 µM BMS-345541 groups were lower in the first examination, so we replicated treatment with intra-articular injections of vehicle, 500 nM and 5 µM BMS-345541 using 7 mice per group for 8 weeks. The number of the mice for the OA analyses decreased for several reasons that were independent of BMS-345541 treatment: water leakage, severe injury due to fighting, and failures during tissue preparations. Finally, the number of mice evaluated was 5, 4, 4, 4, 6, 5 (1st) and 7, 0, 5, 7, 0, 0 (2nd) for vehicle, 50 nM, 500 nM, 5 µM, 50 µM, and 500 µM, respectively. To examine histological changes at the early stage of treatment, we also performed OA surgery in 24 mice, divided them into 6 groups, and analysed them after 2-week treatment.

### Histological analyses

Knees were fixed in 4% paraformaldehyde prepared in phosphate-buffered saline (PBS), decalcified in ethylenediaminetetraacetic acid (EDTA), and embedded in paraffin. To quantify the severity of knee OA, all samples were quantified using the OARSI scoring system (Supplementary Table S2)^18^ by three observers who were blinded to the experimental groups, and the mean value displayed. For quantitative outcome data, Cohen’s d effect size was applied to estimate the overall effect size of BMS-345541 in mice models of OA (<0.2: not clinically relevant; >0.2: small; >0.5: moderate; >0.8: large). For immunohistochemistry, sections were incubated with the following antibodies diluted in blocking reagent: IκBα (1:200; sc-371), Ser32/36 dual phosphorylated IκBα (p-IκBα) (1:100; sc-101713), and Adamts5 (1:500; sc-1349525), all from Santa Cruz Biotechnology (Dallas TX, USA); Hif-2α (1:200, NB100–122; Novus Biologicals, Littleton, CO, USA), and Mmp13 (1:250; MAB13426; Merck Millipore, Darmstadt, Germany). For immunofluorescence, we used a CSA II Biotin-Free Catalyzed Amplification System (K1497, Agilent Technologies). Four representative sections from each treatment group were used to measure the expression level of target protein in articular cartilage (from the superficial layer to the deep layer in the femoral condyle and tibial cartilage) by ImageJ (National Institutes of Health, Bethesda, MD, USA). The percentage of positive cells in control mice was defined as “1”.

### Cell cultures

We obtained samples of human articular cartilage from three individuals undergoing total knee arthroplasty after obtaining written informed consent from the patients. Briefly, less degenerated cartilage was dissected away from the subchondral bone, minced into small pieces, and digested with 3 mg/ml collagenase (Wako; Osaka, Japan) in DMEM/Ham’s F12 (Wako) for 1 h at 37 °C. The digested tissue was then filtered and resuspended with culture medium composed of DMEM/Ham’s F12 supplemented with 10% fetal bovine serum (FBS) (Sigma-Aldrich), and 1% penicillin-streptomycin (Sigma-Aldrich). Cells were seeded into culture dishes and treated with 10 ng/ml IL-1β (Peprotech; Rocky Hill, NJ, USA) in the absence or presence of BMS-345541 (50, 500 nM, 5 µM) for 24 h.

### Quantitative reverse transcription polymerase chain reaction (qRT-PCR)

Total RNA was purified with an RNeasy Mini Kit (Qiagen; Hilden, Germany). One microgram of total RNA was reverse transcribed using ReverTra Ace qPCR RT Master Mix with gDNA Remover (Toyobo; Osaka, Japan). Each PCR contained 1 × THUNDERBIRD SYBR qPCR Mix (Toyobo), 0.3 mM specific primers, and 20 ng cDNA. The mRNA copy number for each specific gene in the total RNA was calculated using a standard curve generated by serially diluted plasmids containing PCR amplicons. Copy number was normalized to rodent total RNA (Thermo-Fisher Scientific; Waltham, MA, USA), with human GAPDH used as an internal control. All reactions were run in triplicate. The primer sequences are shown in Supplementary Table S3.

### Western Blotting

Cells were lysed in M-PER Mammalian Protein Extraction Reagent (Thermo-Fisher Scientific). Equal amounts of protein were subjected to SDS-PAGE using 7.5% to 15% Tris-Glycine gradient gels, and blotted onto PVDF membranes (Bio-Rad Laboratories, Inc.; Hercules, CA, USA). After blocking with 5% skimmed milk, membranes were incubated with primary antibodies against IκBα (1:200), p-IκBα (1:200), HIF-2α (1:500), p65 (1:200, ab7970; Abcam; Burlingame, CA, USA), p- NF-κB p65 (Ser536) (1:200, sc-136548, Santa Cruz Biotechnology), MMP13 (1:200), ADAMTS5 (200:1), COL2 (2000:1, MAB8887; Merck Millipore), or β-ACTIN (1:1,000; Sigma-Aldrich) in Can Get Signal solution (Toyobo). The membranes were then incubated with HRP-conjugated secondary antibody (Promega; Fitchburg, WI, USA), and immunoreactive bands were visualized with ECL plus (GE Healthcare; Buckinghamshire, England, UK) according to the manufacturer’s instructions. The blots were stripped by incubating for 20 min in stripping buffer (2% SDS, 100 mM 2-mercaptoethanol, and 62.5 mM Tris-HCl, pH 6.7) at 50 °C and reblotted with other antibodies. Original images of the immunoblots were shown in Supplementary Fig. S4.

### Luciferase assays

Human primary articular chondrocytes were seeded into the wells of 24-well plates and co-transfected with 500 ng/well the NF-κB reporter vector (pGL4.32[luc2P/ NF-κB-RE/Hygro]) and 2 ng/well pRL-TK (Promega) as an internal control using Lipofectamine 2000 Transfection Reagent (Thermo Fisher Scientific). At 24 h after transfection, cells were treated with 10 ng/ml IL-1β in the absence or presence of BMS-345541. After a further 24 h, luciferase assays were performed using the PicaGene Dual SeaPansy Luminescence Kit (Toyo Ink, Tokyo, Japan) and a GloMax 96 Microplate Luminometer (Promega). Data are presented as the ratio of firefly to Renilla activities.

### Cytotoxicity assay

Cell proliferation and viability were examined using a CCK-8 assay kit (Dojindo; Tokyo, Japan). Human articular chondrocytes were seeded into the wells of a 96-well plate at a density of 5,000 cells/well, and cultured in DMEM/Ham’s F12 containing 10% FBS and 1% penicillin-streptomycin at 37 °C under 5% CO2. On the next day, 10 µL of BMS-345541 solution was added at various concentrations (50 nM, 500 nM, 5 µM, 50 µM, or 500 µM). CCK-8 assay was then performed at 24, 48, 72, or 96 h after the BMS-345541 treatment. CCK-8 substrate (10 µL) was added to each well and incubated for 2 h at 37 °C. We used a plate reader at 450 nm for colorimetric detection (Corona Electric Co.; Ibaraki, Japan).

### Apoptosis assay

Terminal deoxynucleotidyl transferase dUTP nick end labeling (TUNEL) was performed using an *In Situ* Cell Death Detection Kit, Fluorescein (Roche Diagnostics, Mannheim, Germany) to detect DNA fragmentation in cells undergoing programmed cell death. Human articular chondrocytes were cultured on Permanox Lab-Tek® chamber slides (Nalgene Nunc International, Rochester, NY, USA), and treated with each concentration of BMS-345541 for 12 h. Apoptotic cells were then labeled in accordance with manufacturer instructions. Four slides per concentration were used for quantification. Slides were then overlaid with Vectashield® mounting medium containing 4′,6-diamidino-2-phenylindole (DAPI) (Vector Laboratories, Burlingame, CA, USA). Results were presented as TUNEL-positive cells per 100 cells.

### Statistical analyses

For multiple comparisons, statistical significance for differences between groups was determined with a one-way analysis of variance using JMP 13 (SAS Institute, Cary, NC, USA). In one-way ANOVA, the assumptions of the analysis were assessed by the Shapiro–Wilk test of normality and Levene’s test for homogeneity of variance. If the result was significant, statistical analysis was performed with the Kruskal–Wallis and Dunn’s *post hoc* test for unequal variance. ANOVA followed by Dunnett’s *post hoc* test was employed if there was no significant result. For the results presented in all figures, each group was compared with the control. *P* values less than 0.05 were considered significant.

## Electronic supplementary material

Supplementary Tables and Figures

## Data Availability

All data generated or analysed during this study are included in this published article.
