# Supplementary material for: Intra-articular administration of IκBα kinase inhibitor suppresses mouse knee osteoarthritis via downregulation of the NF-κB/HIF-2α axis
Source: Sci Rep. 2018 Nov 7;8:16475. doi: 10.1038/s41598-018-34830-9 (PMC6220282; doi:10.1038/s41598-018-34830-9)
Supplement: Supplementary file 1 — Supplementary Tables and Figures [file 41598_2018_34830_MOESM1_ESM.pdf]

Supplementary Tables and Figures

Intra-articular administration of IκBα kinase inhibitor suppresses mouse knee osteoarthritis via downregulation of the NF-κB/HIF-2α axis

Yasutaka Murahashi[1,3], Fumiko Yano[2], Hiroshi Kobayashi[1], Yuma Makii[1], Kousuke Iba[3], Toshihiko Yamashita[3], Sakae Tanaka[1], and Taku Saito[1]

[1]Sensory & Motor System Medicine, [2]Bone and Cartilage Regenerative Medicine, Graduate School of Medicine, The University of Tokyo, 7-3-1 Hongo, Bunkyo-ku, Tokyo 113-8655, Japan. [3]Department of Orthopaedic Surgery, Sapporo Medical Univ. School of Medicine, S-1, W-16, Chuo-ku, Sapporo, 060-8543, Hokkaido, Japan

		Vehicle	50 nM	500 nM	5 µM	50 µM	500 µM
Body weight (g)	0 w	24.7	23.5	24.4	23.6	25.4	24.6
	8 w	27.8	28	27.4	26.7	27.8	27.8
Rate of weight gain	(%)	12.6	19.1	12.3	13.1	9.4	13.0
P vs Vehicle			0.40	1.00	0.80	0.82	1.00

Supplementary Table S1 Body weights of mice after 8 weeks of BMS-345541 treatment. Data as expressed as means. *P* values were determined by the Kruskal-Wallis & Dunn's post hoc test for unequal variance.

Grade	Osteoarthritic damage
0	Normal
0.5	Loss of Safranin-O without structural changes
1	Small fibrillations without loss of cartilage
2	Vertical clefts down to the layer immediately below the superficial layer and some loss of surface lamina
3	Verical clefts/erosion to the calcified cartilage extending to <25% of the articular surface
4	Vertical clefts/erosion to the calcified cartilage extending to 25-50% of the articular surface
5	Vertical clefts/erosion to the calcified cartilage extending to 50-75% of the articular surface
6	Vertical clefts/erosion to the calcified cartilage extending >75% of the articular surface

Supplementary Table S2 The OARSI scoring system.

Gene Symbol		sequence
MMP13	F	TCCTACAAATCTCGCGGGAAT
	R	GCATTTCTCGGAGCCTCTCA
ADAMTS5	F	TATGACAAGTGCGGAGTATG
	R	TTCAGGCTAAATAGGCAGT
COL2A1	F	GGTGGCTTCCATTTCAGCTA
	R	TACCGGTATGTTTCGTGCAG
GAPDH	F	GAAGGTGAAGGTCGGAGTCA
	R	GAAGATGGTGATGGGATTTC

Supplementary Table S3 Primers used for qRT-PCR.

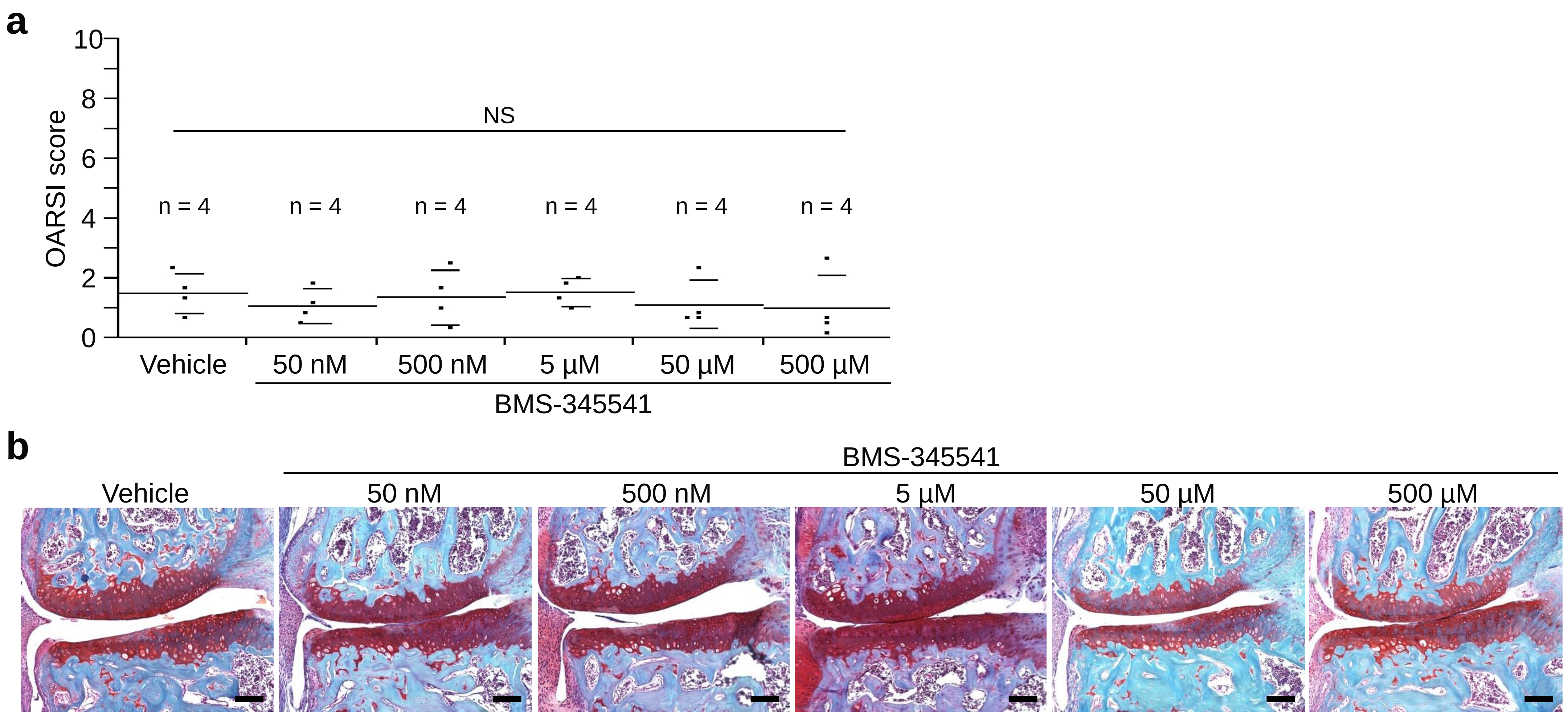

Supplementary Figure S1 | OA development after 2-week BMS-345541 treatment. (**a**) Quantification of OA development using the OARSI score. Symbols represent individual mice; long and short bars show the mean and SD, respectively. NS, not significant. (**b**) Representative safranin O staining from each experimental group. Scale bar, 100 µm.

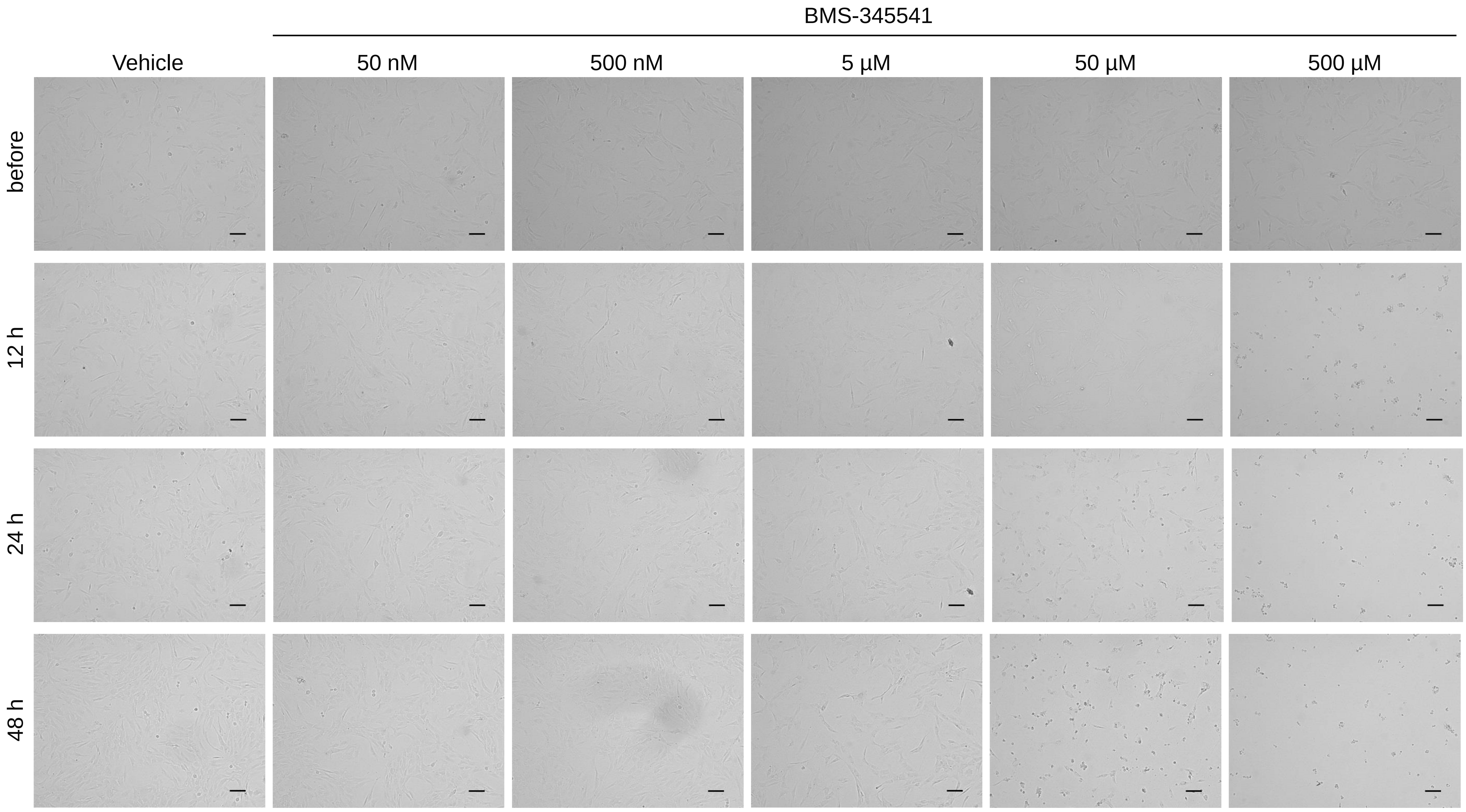

Supplementary Figure S2 | Human articular chondrocytes treated with BMS-345541. Phase-contrast images of human articular chondrocytes treated with vehicle or 50 nM, 500 nM, 5 µM, 50 µM, 500 µM BMS-345541 for 48 hours. Scale bars, 100 µm.

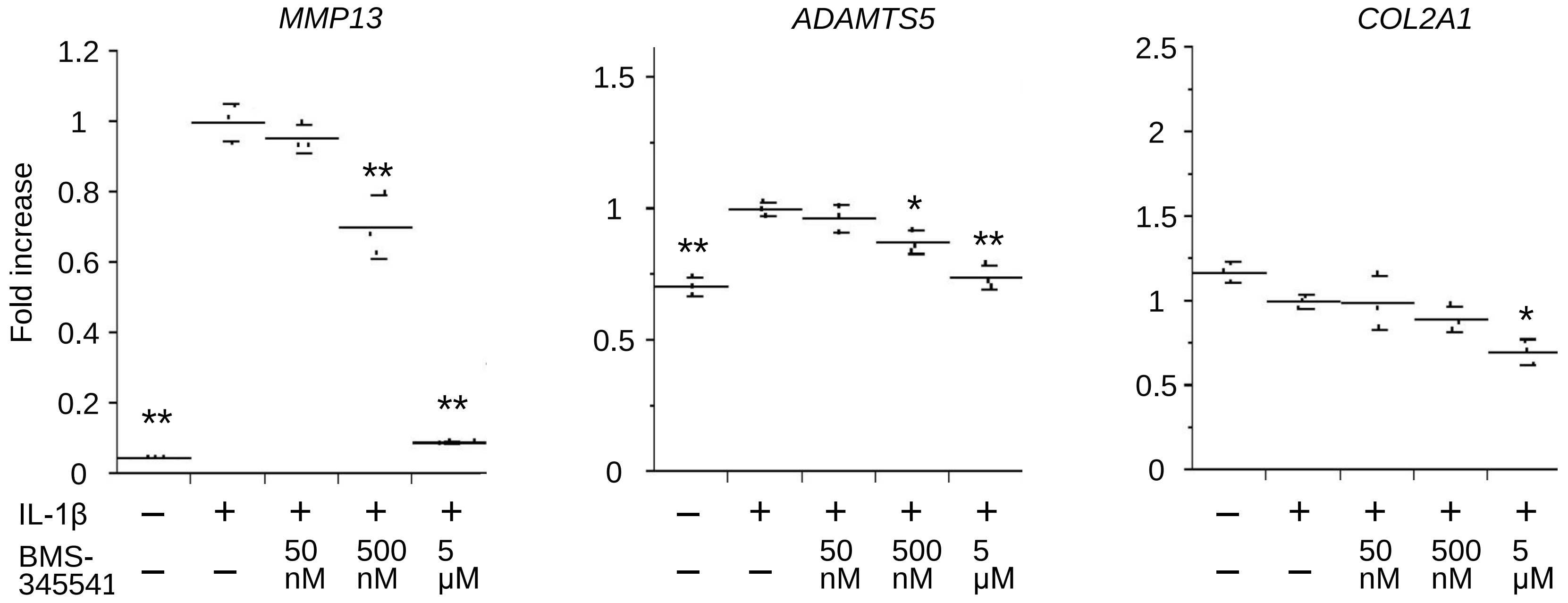

Supplementary Figure S3 | Effects of BMS-345541 in cultured human articular chondrocytes. mRNA levels of *MMP13*, *ADAMTS5*, and *COL2A1* in human primary articular chondrocytes treated with different concentrations of BMS-345541 with or without 10 ng/ml IL-1β. BMS-345541 was added 3 h after IL-1β stimulation, and the cells were cultured for an additional 24 h. The cells obtained from the three individuals showed similar responses, and a representative result is shown. Symbols represent individual points; long and short bars show the mean and SD of three wells per group, respectively. *$P < 0.05$, **$P < 0.0001$ vs IL-1β+/BMS-345541− by the Kruskal–Wallis test and Dunn's *post hoc* test for unequal variance.

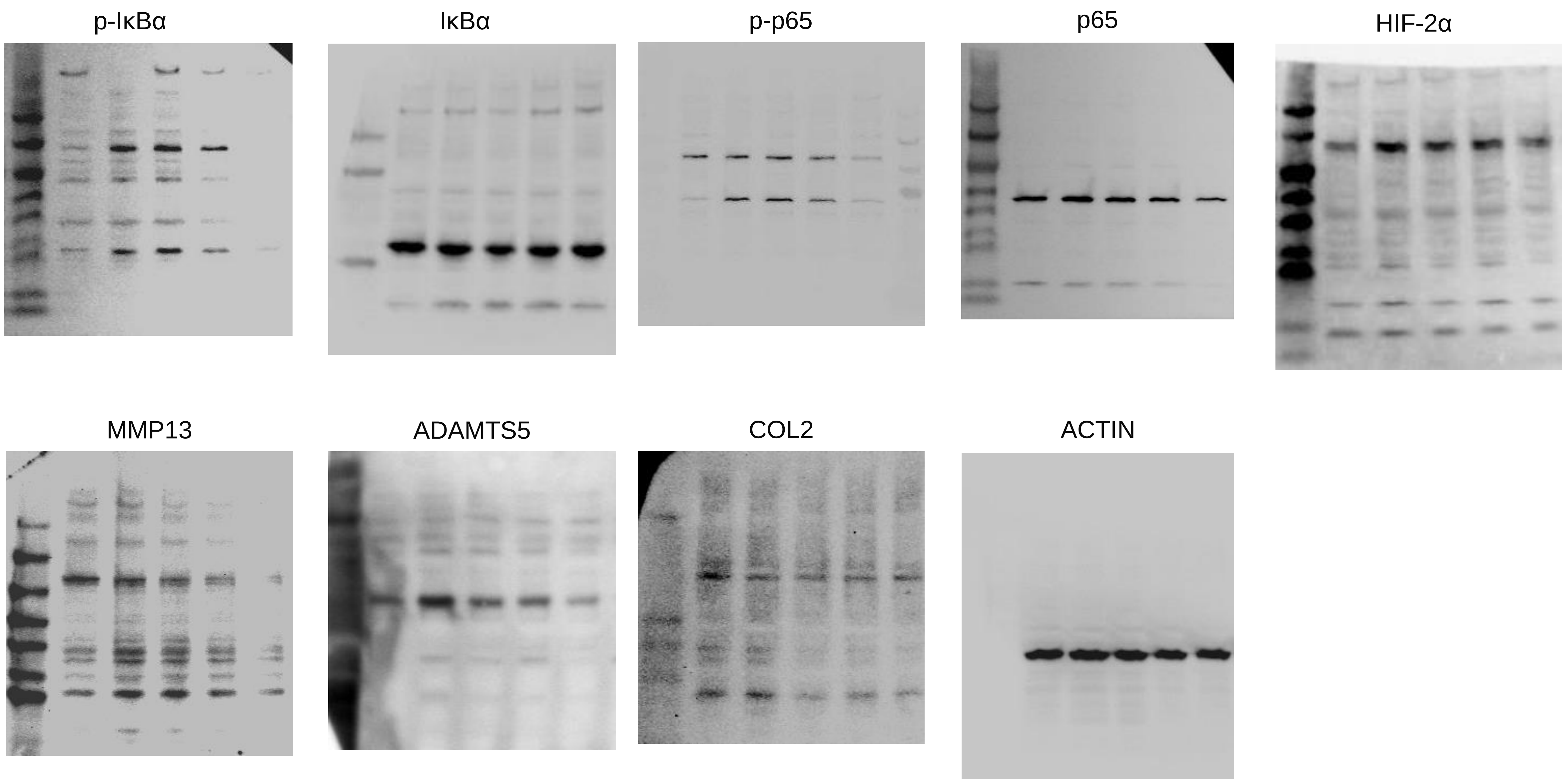

Supplementary Figure S4 | Original images of the immunoblots shown in Fig. 5b.
